# More Expensive, More Attractive? The Effect of Pricing on Gift Evaluation: Differences Between Giver and Receiver

**DOI:** 10.3389/fpsyg.2022.790434

**Published:** 2022-03-30

**Authors:** Ning Liu, Yu Lou, Xinyu Wang, Shouxin Li

**Affiliations:** School of Psychology, Shandong Normal University, Jinan, China

**Keywords:** gift-giving, price, desirability, feasibility, psychological distance, gift evaluation, giver-receiver difference

## Abstract

The present research explored differences in gift evaluation between gift givers and receivers. Three studies were conducted to test how the pricing influenced the gift evaluations of givers and receivers, and whether the price-quality and price-monetary sacrifice inferences were the underlying mechanisms. The results showed that: givers evaluated high-priced gifts as better than low-priced gifts, whereas receivers evaluated low-priced gifts as better than high-priced gifts; price-quality inference mediated the effect of pricing on gift evaluations, but only for givers. Furthermore, the effect of pricing on gift evaluation was moderated by the gift type: givers evaluated the high-priced gift as better only for the desirable gift (but not for the feasible gift); receivers evaluated the low-priced gift as better only for the feasible gift (but not for the desirable gift). The results demonstrate the effect of pricing on gift evaluation and could contribute to understanding the differences between givers’ and receivers’ perception of what a “good gift” is, and the underlying psychological mechanisms.

## Introduction

Gift-giving can improve social bonds between givers and receivers (Schwartz, 1967; Belk, 1979). Givers try to please receivers with a well-received gift, but too often give gifts that receivers do not want. For example, research has shown that gift receivers appreciated gifts that they requested more than “thoughtful and considerate” gifts that they did not explicitly request (Gino and Flynn, 2011). Gift-giving can be very challenging since there are discrepancies between givers’ and receivers’ perspectives on what constitutes a “good gift” (e.g., Galak et al., 2016; Givi et al., 2021). For example, givers prefer desirable gifts more than feasible gifts, while receivers exhibit no such preference (Baskin et al., 2014). Desirability refers to the central aspect and value of a gift’s end state, such as food quality of a restaurant; feasibility refers to the non-essential aspect and the means of achieving that end state, such as the convenience of getting to a restaurant (Liberman and Trope, 1998). Previous research has shown that social distance may drive discrepancies between gift givers and receivers (Baskin et al., 2014), because givers consider their own preferences in addition to the receiver’s preferences, therefore evaluating the gift from a farther social distance. By contrast, receivers only consider their own preference, thereby evaluating gifts from a closer social distance. Consequently, the asymmetric social distance affects the balance between desirability and feasibility, with givers placing more weight on the former and receivers on the latter (Baskin et al., 2014).

We aimed to examine how the gift price, the extrinsic value label, influenced givers’ and receivers’ gift evaluations. Particularly, we were interested in the question of why givers seem to prefer high-priced gifts, believing them to be “more expensive, more attractive.” To our knowledge, little research has directly investigated the effect of gift pricing on both givers’ and receivers’ gift evaluation; instead, it has predominantly focused on the feelings of the receiver, while ignoring the evaluation of the gift itself. For example, Wang and Van der Lans (2018) indicated that reduced price sensitivity is the reason why givers tend to pay more than the receivers’ valuation, as givers use price to signal the importance of their relationship with receivers. Additionally, Flynn and Adams (2009) identified a discrepancy between gift-givers and gift-receivers regarding the relationship between gift price and the receiver’s feeling of appreciation. The giver believed that a high-priced gift expresses a higher level of thoughtfulness that should improve the receiver’s appreciation, but the receiver did not share that perspective. However, in this study, we focused on the gift evaluation and assumed that price-quality inference was the psychological mechanism behind the phenomenon of “more expensive, more attractive.”

Previous price-related research also seemed to regard price as the monetary cost, consequently assuming that the more the givers spent on the gift, the more the receivers felt appreciated, and the closer the relationship was between the givers and the receivers (Flynn and Adams, 2009; Wang and Van der Lans, 2018). However, the literature on price perception indicates that price play dual roles indicating not only the monetary sacrifice but also perceived quality (Erickson and Johansson, 1985; Volckner, 2008). Furthermore, Bornemann and Homburg (2011) showed that psychological distance can alter the weight that consumers attach to the price roles. The price-perceived quality relationship is more pronounced under the condition of far psychological distance (vs. near psychological distance); the price-perceived sacrifice relationship is more pronounced under the condition of near psychological distance (vs. far psychological distance). In addition, prior research shows that consumers’ price-quality inference tends to be enhanced when the psychological distance is far (Yan and Sengupta, 2011). Given the effect of psychological distance on the dual roles of price and the findings that giver and receiver have different psychological distance to the gift (Baskin et al., 2014), we proposed that givers with a far psychological distance would be more inclined than receivers to use price to infer quality, resulting in givers believing that high price indicates good quality, and good quality indicates a good gift.

### Psychological Distance and Level of Construal

Psychological distance is a subjective sense of how close or far away something is from the self, here and now. It includes four dimensions: temporal distance, social distance, spatial distance, and hypothetical distance. Construal level theory argues that psychological distance influences mental representation and judgment, where people adopt increasingly higher levels of construal to represent objects as psychological distance increases. Compared with low-level construal, high-level construal is a relatively abstract, coherent, and superordinate mental representation. Construal level can influence desirability and feasibility weightings, specifically, far psychological distance increased people’s focus on desirability, whereas close psychological distance increased their focus on feasibility (Trope and Liberman, 2010).

Consistent with the construal fit view of psychological distance and desirability-feasibility, Liberman and Trope (1998) showed that as the temporal distance from an activity (e.g., going to a restaurant) increased, the attractiveness of the activity depended more on its desirability (e.g., food quality) and less on its feasibility (e.g., the convenience of getting to the restaurant). Similar results have been reported for other dimensions of psychological distance, such as spatial distance (Fujita et al., 2006), hypothetical distance (Todorov et al., 2007), and social distance (Liviatan et al., 2008). In addition, differences in self-other decision-making have been identified as important social distance exemplars. Previous studies have found that decisions for others are made from a farther psychological distance than decisions for self. For example, individuals making decisions for others tended to weigh central attributes more than non-central attributes, which is consistent with construal level theory (Kray and Gonzalez, 1999; Kray, 2000). Moreover, individuals who decided for others tended to weigh desirability more than feasibility than those who decided for themselves (Xu and Xie, 2011; Lu et al., 2013).

In a gift-giving context, the desirability and feasibility trade-off discrepancy between givers and receivers can also be explained by social distance. Recent research showed that givers construed gifts from a further psychological distance (high-level construal) and gave more weight to desirability attributes than feasibility attributes (Baskin et al., 2014). Given the psychological distance discrepancy between gift givers and receivers, we expected that givers would weight desirability more than feasibility and thereby evaluate desirable gifts as better; receivers would weight feasibility more than desirability and thereby evaluate feasible gifts as better.

### Psychological Distance and Price Perception

Price perception is an interesting area for both consumer behavior and social psychology researchers. According to classical economic theory, price is an indicator of monetary sacrifice or cost. However, a growing body of consumer behavior research indicates that price also acts as a product quality indicator, which is known as the price-quality inference (Rao and Monroe, 1988). The indicators of perceived quality and perceived monetary sacrifice are regarded as the dual role of price (Volckner, 2008; Bornemann and Homburg, 2011). In contrast to the traditional economic research perspective, consumer behavioral research focuses on when and why prices act as quality indicators or monetary sacrifice indicators.

Price can be regarded as an extrinsic product value label, and the extent to which people may use it as an indicator of quality is influenced by culture. Previous studies have identified cultural factors that could influence individual’s price-quality inference. For example, power distance refers to the extent to which a culture has respect for authority (e.g., China is high in power distance). Compared with individuals in low power-distance societies, individuals in high power-distance societies have a greater tendency to use price information to infer quality (Lalwani and Forcum, 2016). Similarly, Lalwani and Shavitt (2013) examined the effect of cultural self-construal, the extent to which the self is defined independently of others or interdependently with others. The results showed that, compared with independent cultural self-construal, individuals with interdependent cultural self-construal were more likely to use price information to judge quality, since interdependent people have a more holistic style of thinking and are more likely to perceive positive interrelations between price and product quality (Lalwani and Shavitt, 2013). These findings demonstrated cultural differences in price-quality inference, highlighting how price influences gift evaluation in different cultures, for example, in Chinese culture, where high power distance and interdependent self are predominant, consumers are more likely to infer the quality of a product from its price, that is, the higher the price, the higher the quality.

Apart from the influence of culture, mindset can also determine the role of price as an indicator of quality or an indicator of monetary sacrifice. Sengupta (2011) demonstrated that consumers’ reliance on price for quality inferences was enhanced under high construal conditions (i.e., far psychological distance). Additionally, researchers found that how individuals used price information was also influenced by their goal orientation (i.e., promotion or prevention focus). Consumers with a promotion focus might see high price as an indicator of good quality, while those with a prevention focus might regard high price as an indicator of monetary sacrifice (Lin et al., 2007). Particularly, psychological distance influences the dual roles of price. Previous studies found that consumers’ reliance on price for quality inferences was enhanced when the judgment was psychologically distant (from both a temporally and a socially distant perspective). In other words, individuals who are psychologically distant from a product may prefer higher-priced products because the higher price indicates good quality, while from a temporally proximal perspective, the price-perceived sacrifice relationship may be more pronounced (Bornemann and Homburg, 2011). In light of this, we proposed that gift giver and receiver roles would influence the price-quality inference and gift evaluation, where, even for an identical gift, pricing would have a different effect on givers and receivers. Specifically, compared with receivers, givers who have a greater psychological distance from gift would evaluate the high-priced gift as better than the low-priced gift. Moreover, the underlying mechanism was that givers were more likely to infer quality from price.

### The Desirable-Feasible Gift Types as the Boundary Condition

Additionally, we proposed that the effect of pricing on gift evaluation had the boundary condition of gift type, specifically, givers would use price as a gift evaluation clue for desirable gifts but not for feasible gifts. Previous research found an asymmetric preference for the desirable-feasible gift type, with givers preferring a desirable gift, and receivers preferring a feasible gift (Baskin et al., 2014). More importantly, there is also evidence that it is the stronger focus on desirability (vs. feasibility) that leads consumers to view high price as an indicator of high quality, when the psychological distance is far (Bornemann and Homburg, 2011). Given that a desirable gift description emphasizes the high desirability and low feasibility of the gift (which would brought the consumers more thought about the desirability); and the feasible gift description emphasizes the high feasibility and the low desirability of the gift (which would brought the consumers more thought about the feasibility) (Bornemann and Homburg, 2011), therefore, we expect consumers to view high price as high quality only for the desirable gift but not for the feasible gift. For example, for a restaurant with delicious food but inconvenient to go to, a higher price could indicate a better restaurant; while for a conveniently nearby restaurant but without delicious food, a higher price would not necessarily mean a better restaurant.

### Overview of the Present Research

Three studies were conducted to test how pricing influenced the gift evaluations of givers and receivers, and whether the price-quality and price-monetary sacrifice inferences were the underlying mechanisms. In Study 1, we investigated the effects of the gift-giving role (i.e., giver and receiver) and the gift type (i.e., desirable gift and feasible gift) on gift evaluation; and aimed to provide evidence for the psychological distance difference between givers and receivers by identifying preference asymmetries. In Study 2, we explored the effects of the gift-giving role and pricing on gift evaluation, and further examined the mediating effects of price roles (i.e., perceived quality and perceived monetary sacrifice). Study 3 was designed to identify the boundary condition of the effect of pricing on gift evaluation by examining the moderating effect of gift type (i.e., desirable gift and feasible gift). Overall, the present research could contribute to understand the differences between givers’ and receivers’ idea of what constitutes a “good gift,” and the underlying psychological mechanism behind the differences. It could especially help understand why givers tend to prefer high-priced gifts.

## Study 1: Giver-Receiver Preference Asymmetries on Gift Type

The aim of Study 1 was twofold: to examine desirable gift and feasible gift preference differences between givers and receivers; and provide evidence for differences in psychological distance between givers and receivers by identifying preference asymmetries.

### Methods

#### Design and Participants

In all three studies that adopted the similar experiment design, we determined the sample size by utilizing G*Power software (Faul et al., 2007). Because there was no viable effect size estimate of interaction between gift-giving role and gift type in prior studies, we conducted a power analysis with the medium effect size estimate of 0.30 (*f* = 0.30). The analysis for a two factor between-subjects design suggested that 119 participants would be needed to achieve 0.90 power (1-β) at a 0.05 alpha level (α = 0.05).

A total of 120 Chinese college students were recruited for Study 1 (62 males and 58 females; *M*_*age*_ = 20.38 years, *SD* = 1.35). Participants were randomly assigned to a 2 (gift-giving role: giver vs. receiver) × 2 (gift type: feasible gift vs. desirable gift) between-subjects design. Each participant was instructed to imagine that they were either giving or receiving a gift and to evaluate the gift.

#### Procedure and Materials

First, to help participants engage in the gift-giving or receiving scenarios, they were asked to recall a friend and write down his or her name. They then recalled the most recent scene of giving or receiving gifts according to their roles and wrote down what the gift was. Second, participants in the gift-giver role were asked to imagine that “the friend’s birthday is coming soon and you’ll prepare a gift for him or her,” while participants in the gift-receiver role were asked to imagine that “your birthday is coming soon and the friend prepared a gift for you.” After that, product (i.e., earphones) descriptions of desirable gift or feasible gift, including detailed information about price and basic features, were presented. The price of both the desirable and feasible gifts was 79 RMB (approximately $12), which was set according to the pretest by averaging the willing-to-pay price of the target product. The basic feature descriptions were the same for both gift types.

The gift types were manipulated using consumer comments regarding the product advantages and disadvantages; the feasible gift was described as “convenient to maintain, durable (does not require extra care), but not excellent sound quality” (high feasibility-low desirability), and the desirable gift was described as “excellent sound quality, but not convenient to maintain, and requires good care” (high desirability-low feasibility). The results of a pretest showed that the product descriptions were successfully manipulated.^1^ Next, participants were asked to evaluate the attractiveness of the gift on a 7-point scale, including the degree to which they liked the product as a gift, how good the gift was, how appropriate it was, and how positive it was (Baskin et al., 2014). Third, participants were asked to indicate how desirable and how feasible the earphones were on a 7-point scale, which was used as a manipulation check. Afterward, two questions were asked: (a) “what was the price of the earphones?” (b) “what role in the gift-giving scenario did you play?” These items were designed to check whether participants completed the questionnaire with full attention. Last, demographic information was collected, and the participants were debriefed and thanked.

### Results

SPSS 25.0 was used to analyze the data.

#### Manipulation Check

Participants perceived the feasible gift as more feasible (*M* = 5.12, *SD* = 1.32 vs. *M* = 3.40, *SD* = 1.40), *t*(118) = 6.91, *p* < 0.001, *d* = 1.26; and perceived the desirable gift as more desirable (*M* = 4.93, *SD* = 1.45 vs. *M* = 3.55, *SD* = 1.28), *t*(118) = 5.54, *p* < 0.001, *d* = 1.01, which showed that the gift type manipulation was successful.

#### Gift Evaluation

**FIGURE 1:**
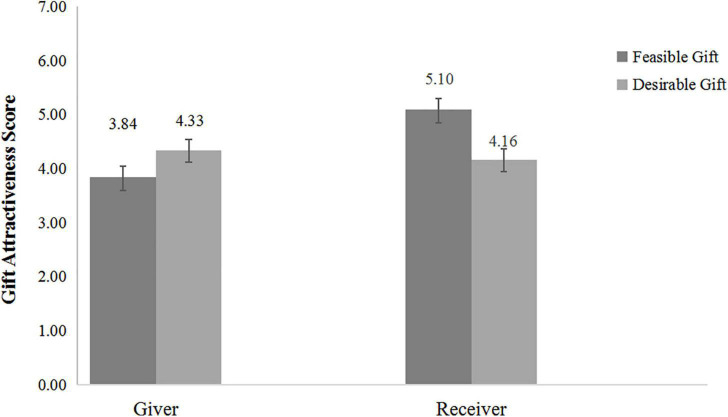
Study 1: Gift evaluation scores of desirable gifts and feasible gifts.

The average of the four gift-evaluation scores was the dependent variable (α = 0.91). A 2 (gift-giving role: giver vs. receiver) × 2 (gift type: feasible gift vs. desirable gift) ANOVA on the score revealed two significant effects: the main effect of gift-giving role was significant, where the receiver’s gift evaluation score was higher than the giver’s, *F*_(1, 116)_ = 6.20, *p* = 0.014, η*_*p*_*^2^ = 0.051; the main effect of gift type was not significant, *F*_(1, 116)_ = 1.09, *p* = 0.298. The preference asymmetry hypothesis was supported by the significant interaction between gift-giving role and gift type, *F*_(1, 116)_ = 10.56, *p* = 0.002, η*_*p*_*^2^ = 0.083, as shown in Figure 1. The simple effect analysis indicated that givers thought the desirable gift (*M* = 4.33, *SD* = 1.35) was more attractive than the feasible gift (*M* = 3.84, *SD* = 1.11), although it was not significant (*p* = 0.12). For receivers, the feasible gift (*M* = 5.10, *SD* = 1.16) was significantly more attractive than the desirable gift (*M* = 4.16, *SD* = 1.17), *p* = 0.003, *d* = 0.81.

### Discussion

As expected, Study 1 showed that givers rated desirable gifts higher than feasible gifts, while receivers rated feasible gifts higher than desirable gifts. Specifically, givers preferred the desirable gift because they cared more about the sound quality of the earphones than their cost of maintenance; whereas receivers, as the real users of the earphones, preferred the feasible gift because they cared more about the maintenance costs than the sound quality. These findings were similar to those of research (Baskin et al., 2014). Furthermore, together with Baskin et al. (2014) findings, the pattern of preference asymmetries also provided evidence that givers’ and receivers’ psychological distance was contrasting (further and closer, respectively).

Additionally, similar to the findings of previous research (Baskin et al., 2014), Study 1 revealed that givers showed a preference for desirable gifts; however, unlike in previous research, in Study 1 this preference was not significant. The reason might be that the chosen price (79 RMB) of the target gift influenced the givers’ evaluation of the desirable gift. In Baskin et al.’s (2014) research, price information was not provided to participants, but in our study, the price was clearly set for both the desirable gift and the feasible gift, to ensure a realistic product description. The price of 79 RMB might not have been high enough to match the “desirable gift” description, which may have led to the givers’ lower evaluations for desirable gift than what was expected. To address the question of how price influences gift evaluation, and the difference between givers and receivers, we directly investigated the effect of pricing and gift-giving roles on gift evaluation in Study 2.

## Study 2: The Effect of Pricing on Gift Evaluation and the Mechanism

The objective of Study 2 was to explore gift-giving role differences in the effect of pricing on gift evaluation, and further explore the psychological mechanism behind such difference. We expected givers to infer quality from the price because of their far psychological distance, and they would think that high-priced gifts were more attractive than low-priced ones, even for identical gifts. By contrast, receivers would not use price as an indicator of quality, due to their close psychological distance; and they would not differentiate the monetary costs of the high and low price gifts either, since it was not them who actually purchased the gifts.

### Methods

#### Design and Participants

Similar with Study 1, a total of 120 Chinese college students were recruited for this study (56 males and 64 females; *M*_*age*_ = 20.57 years, *SD* = 1.43), who were randomly assigned to a 2 (gift-giving role: giver vs. receiver) × 2 (gift price: low vs. high) between-subjects design. Each participant was instructed to imagine that they were either giving or receiving a gift, and to evaluate the attractiveness of a high-priced or low-priced gift, according to their experimental conditions.

#### Procedure and Materials

The procedure of Study 2 was almost the same as Study 1. Participants were first instructed to recall a friend and write down his or her name. They then recalled the most recent scene of giving or receiving gifts according to their role and wrote down what the gift was, after which they were asked to imagine the “birthday gift-giving or receiving” scenario just as in Study 1. The exact same product (earphones) descriptions were presented to the participants in all experimental conditions. The important features were described as “elegant and natural design with a metal outer case, impressive bass quality, restoring voice accurately by a 9 mm loudspeaker, and ergonomic design.” The gift earphones’ prices were manipulated depending on the experimental condition. Participants in the low-price condition were informed that the earphones cost 39 RMB (approximately $6), while in the high-price condition participants were told that the earphones cost 119 RMB (approximately $17). The high-price and low-price were determined according to a pretest, which asked participants (*N* = 27) to read the product description and indicate the highest and lowest price they were willing to pay for the target product. Participants then evaluated the earphones as gifts on a 7-point scale. To explore the psychological mechanism behind the effect of pricing, participants were instructed to evaluate the perceived quality (i.e., the product appears to be of good quality; the product appears to be reliable, α = 0.87) and the perceived monetary sacrifice (i.e., the advertised price is very high; the product is very expensive, α = 0.81) on a 7-point scale (Bornemann and Homburg, 2011). As the manipulation check of gift price, participants were also asked to rate how costly the earphones were on a 7-point scale. Then the same attention check questions were asked as in Study 1. Finally, demographic information was collected, and all participants were debriefed and thanked.

### Results

#### Manipulation Check

The earphones were perceived as more expensive by participants in the high price condition (*M* = 4.63, *SD* = 1.13) than those in the low price condition (*M* = 3.66, *SD* = 1.10),*t*(117) = 4.731, *p* < 0.001, *d* = 0.87, indicating a successful manipulation of the gift price.

#### Gift Evaluation

**FIGURE 2:**
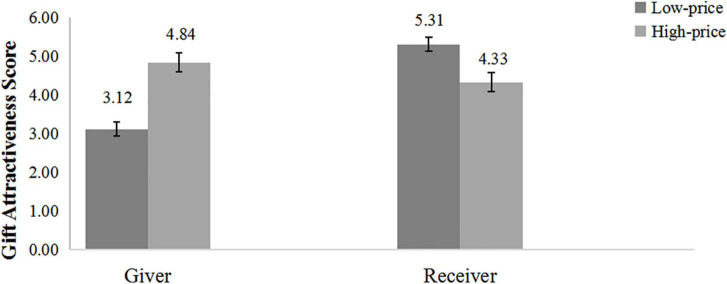
Study 2: Gift evaluation scores of high-price gifts and low-price gifts.

The average of the four gift-evaluation scores was the dependent variable (α = 0.92). A 2 (gift-giving role: giver vs. receiver) × 2 (gift price: low vs. high) ANOVA on gift evaluation score yielded two significant effects. The significant main effect of gift-giving role, where receivers’ evaluation scores were higher than givers’ scores, *F*_(1, 116)_ = 13.25, *p* < 0.001, η*_*p*_*^2^ = 0.10; the main effect of pricing was not significant, *F*_(1, 116)_ = 2.63, *p* = 0.108. The preference asymmetry regarding price was supported by a significant interaction between participant role and gift price, *F*_(1, 116)_ = 34.09, *p* < 0.001, η*_*p*_*^2^ = 0.23, as shown in Figure 2. Further simple effect analysis indicated that givers evaluated the high-priced gift as significantly better than the low-priced gift (*M*_high–price_ = 4.84, *SD*_high–price_ = 1.37, *M*_low–price_ = 3.12, *SD*_low–price_ = 1.01, *p* < 0.001, *d* = 1.43). However, receivers evaluated the low-priced gift as significantly better than the high-priced gift (*M*_high–price_ = 4.33, *SD*_high–price_ = 1.31, *M*_low–price_ = 5.31, *SD*_low–price_ = 1.34, *p* = 0.003, *d* = 0.74).

#### Mediation Analysis

Averaging the two perceived quality (PQ) scores created a PQ score (α = 0.87); and averaging the perceived monetary sacrifice (PS) scores created a PS score (α = 0.81). The dual mediation analysis was conducted separately for givers and receivers using a bootstrapping procedure (model 4; with 5,000 re-samples) to examine the indirect effect of gift price on gift evaluation through PQ and PS (Preacher and Hayes, 2004).

**FIGURE 3:**
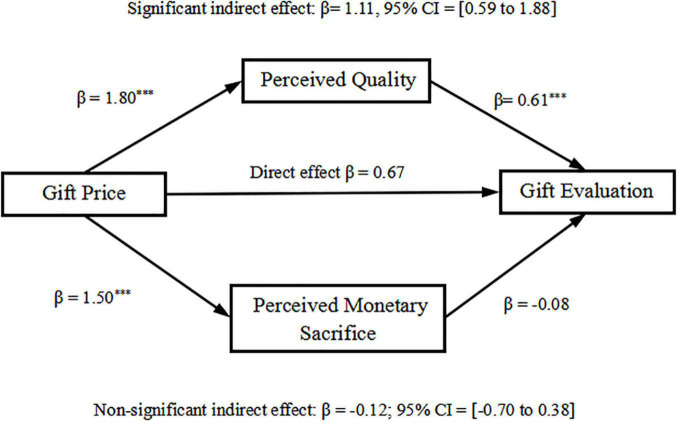
Mediation of PQ and PS between gift price and gift evaluations for givers (***p < 0.001).

For givers, the results showed that the indirect effect of pricing through PQ on gift evaluation was significant (β = 1.11, 95% CI = 0.59–1.88); whereas the indirect effect of pricing through PS on gift evaluation was not significant (β = −0.12, 95% CI = −0.70 to 0.38). Moreover, the direct effect of pricing on gift evaluation was not significant (β = 0.67, *p* = 0.10; 95% CI = −0.12 to 1.46). This result indicated that, for givers, the effect of gift price on gift evaluation was mediated by PQ, as shown in Figure 3. Specifically, PQ could be predicted by price (β = 1.80, *p* < 0.001; 95% CI = 1.27 to 2.33), and could predict the gift evaluation (β = 0.61, *p* = 0.0001; 95% CI = 0.31 to 0.92); while PS could be predicted by price (β = 1.50, *p* < 0.001; 95% CI = 1.05 to 1.94), but could not predict gift evaluation (β = −0.08, *p* = 0.65; 95% CI = −0.44 to 0.28).

For receivers, the result showed that the indirect effect of pricing through PQ on gift evaluation was not significant (β = −0.009, 95% CI = −0.40 to 0.38); the indirect effect of pricing through PS was not significant either (β = 0.09, 95% CI = −0.01 to 0.37); and the direct effect of pricing on gift evaluation was significant (β = −1.06, *p* = 0.0001; 95% CI = −1.57 to −0.55). In addition, PQ could not be predicted by price (β = −0.02, *p* = 0.96; 95% CI = −0.73 to 0.70) but could predict the gift evaluation (β = 0.54, *p* < 0.001; 95% CI = 0.31 to 0.78); PS could not be predicted by price (β = 0.45, *p* = 0.16; 95% CI = −0.18 to 1.08) and could not predict the gift evaluation (β = 0.21, *p* = 0.12; 95% CI = −0.06 to 0.47). These results indicated that, for receivers, the effect of pricing on gift evaluation was not mediated by either PQ or PS.

### Discussion

The results supported the hypothesis of “more expensive, more attractive” for givers but not for receivers, who even considered the less expensive gift as more attractive. Furthermore, the effect of pricing on gift evaluations for givers was mediated by the price-quality inference. The mediation result were consistent with previous research, in which the researchers found that the quality perceptions could mediate the influence of price on product evaluation under the condition of distant social distance (Bornemann and Homburg, 2011). Additionally, Bornemann and Homburg (2011) also found that distant social distance might lead to a stronger focus on desirability compared to feasibility, which mediated the price-quality inference, in other words, consumers regarded high price as an indicator of desirability (high quality) rather than feasibility (high sacrifice). Regarding the perceived monetary sacrifice, the other role of price, givers regarded higher price as more costly, however, their gift evaluations were not influenced by perception of the monetary sacrifice, which suggested that givers were sensitive to the cost of the gift, but they were willing to bear such cost for a high-quality gift.

Receivers, with a closer psychological distance, regarded price neither as an indicator of perceived quality nor as an indicator of perceived monetary sacrifice, because they did not need to bear the monetary cost of the gift, although previous research showed that monetary cost perceptions could mediate the influence of price on product evaluation under the condition of proximal social distance (Bornemann and Homburg, 2011). Moreover, the results showed that receivers evaluated low-priced gifts as better than high-priced gifts, and the direct effect of pricing on gift evaluation for receivers was significant, suggesting that receivers evaluated low-priced gifts as better, not through the indirect path of perception of quality and monetary cost.

The finding that receivers preferred low-priced gifts is interesting, and might be explained from the perspective of cost. First, prior research had shown that, compared with givers, receivers were more sensitive to the use cost (i.e., feasibility of the gift) and the behavioral cost (i.e., time, mental, and physical efforts givers undergo to choose the gift) rather than the monetary cost, resulting in the preference for gifts with high feasibility and high behavioral cost (Robben and Verhallen, 1994; Baskin et al., 2014). In this case, a low-priced gift was preferred, not for its lower monetary cost (since receivers were not sensitive to this) but for its lower use cost, since compared with a low-priced gift, a high-priced gift usually requires extra care and maintenance from the user. Second, gift-giving could be taken as an exchange process, especially for a birthday occasion, where reciprocity is the normal rule, requiring an adequate or, at least, equal value return gift (Mauss, 1970; Schiffman and Cohn, 2009). Therefore, a high-priced gift can also imply a level of burden, which could be regarded as the emotional cost of the gift, and givers usually underestimate how uncomfortable receivers feel receiving a gift without reciprocating (Givi, 2021). Consequently, receivers might also take low price as an indicator of low emotional cost, which could be regarded as kindness or “thoughtful consideration for friends,” that is, because givers feel close to receivers, they do not want to give them an expensive but burdensome gift (Gergen et al., 1975; Sherry, 1983; Cotterell et al., 1992). Both explanations shared the same view that receivers took the price as an indicator of cost (use cost and behavioral cost) and they preferred the low-cost gift.

Study 2 demonstrated the opposite effect of pricing on gift evaluation for different gift-giving roles: givers evaluate high-price gifts as better than low-price gifts; whereas receivers evaluate low-price gifts as better than high-price gifts. In Study 3, we aimed to identify the boundary condition of the pricing effect and compare the pricing effect patterns between giver and receiver by exploring the moderating effect of desirable-feasible gift types in two separate experiments.

## Study 3A: The Boundary Condition of Desirable-Feasible Gift Types for Givers

The objective of Study 3A was to identify the boundary condition of the pricing effect for givers by exploring the moderating role of desirable-feasible gift type. As discussed in the Introduction, we expected givers to evaluate the high-priced gift as better than the low-priced one, but only for the desirable gift.

### Methods

A total of 122 Chinese college students participated; one participant’s data were excluded for not completing the full task, leaving 121 participants for the analyses (56 males, 63 females, and 2 who did not report gender; *M*_*age*_ = 20.79 years, *SD* = 1.68). Participants were randomly assigned to a 2 (gift price: low vs. high) × 2 (gift type: feasible gift vs. desirable gift) between-subjects design and instructed to imagine that they were giving a gift, and to evaluate the gift.

The procedure was similar to that of Study 1 and 2, except that there were four different earphone product descriptions: high-priced desirable gift, low-priced desirable gift, high-priced feasible gift, and low-priced feasible gift. Participants evaluated the earphones after reading the descriptions of desirable gift or feasible gift which were exactly the same as Study 1’s. Subsequently, they were asked to indicate how desirable or how feasible the earphones were on a 7-point scale, and to rate how costly the earphones were on another 7-point scale as a manipulation check. Attention check questions were also asked. Finally, demographic information was collected, and the participants were debriefed and thanked.

### Results

#### Manipulation Check

Participants perceived the feasible gift as more feasible (*M* = 4.61, *SD* = 1.15 vs. *M* = 3.53, *SD* = 1.28), *t*(119) = 4.88, *p* < 0.001, *d* = 0.89. Similarly, they perceived the desirable gift as more desirable (*M* = 4.38, *SD* = 1.26 vs. *M* = 3.16, *SD* = 0.94), *t*(119) = 5.91, *p* < 0.001, *d* = 1.10, showing that the gift type manipulation was successful. Moreover, the earphones were perceived as more expensive by participants in the high-price condition (*M* = 4.48, *SD* = 1.17) than those in the low-price condition (*M* = 2.97, *SD* = 0.82), *t*(119) = 8.23, *p* < 0.001, *d* = 1.49, showing that the gift price manipulation was also successful.

#### Gift Evaluation

**FIGURE 4:**
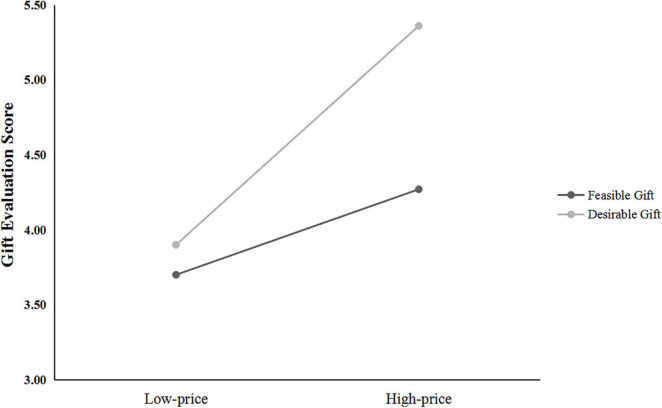
Study 3A: Gift evaluation scores of gift givers.

The average of the four gift-evaluation scores was the dependent variable (α = 0.93). A 2 (gift price: low vs. high) × 2 (gift type: feasible gift vs. desirable gift) ANOVA on the score yielded three significant effects. First, there was a significant main effect for gift price, with the high-priced gift evaluation scored higher than the low-priced gift, *F*_(1, 117)_ = 12.31, *p* = 0.001, η*_*p*_*^2^ = 0.10. Second, the main effect of gift type was also significant, and the desirable gift evaluation score was higher than that of the feasible gift, *F*_(1, 117)_ = 8.61, *p* = 0.004, η*_*p*_*^2^ = 0.07. More importantly, for givers, the interaction between gift type and gift price was significant, *F*_(1, 117)_ = 5.80, *p* = 0.018, η*_*p*_*^2^ = 0.05, as shown in Figure 4. The simple effect analysis showed that for the desirable gift, the high-priced gift (*M* = 5.34, *SD* = 1.18) was significantly better than the low-priced gift (*M* = 3.93, *SD* = 0.95, *p* < 0.001, *d* = 1.32), which replicated the result of Study 2. However, the gift evaluation difference for the feasible gift was not significant (*M*_high–price_ = 4.07, *SD*_high–price_ = 1.55, *M*_low–price_ = 3.81, *SD*_low–price_ = 1.49, *p* = 0.458). Notably, as shown in Table 1, givers rated the high-priced desirable gift as the most attractive one, which was consistent with our prediction.

**TABLE 1:** Gift evaluation in Study 3 (*M* ± *SD*).

Gift-giving Role	Gift price	Gift type	
		
		Desirable gift	Feasible gift
Givers	High-price	5.34 ± 1.18	4.07 ± 1.55
	Low-price	3.93 ± 0.95	3.81 ± 1.49
Receivers	High-price	5.01 ± 1.13	4.48 ± 1.25
	Low-price	4.70 ± 1.30	5.23 ± 1.14

The results supported our prediction that the consumers evaluated high-priced gifts as better than low-priced gifts only for the desirable gift but not for the feasible gift. They took high price as high quality only for the desirable gift, and consequently, a high-priced desirable gift was their choice as the best gift.

## Study 3B: The Boundary Condition of Desirable-Feasible Gift Types for Receivers

The objective of Study 3B was to identify the boundary condition of the pricing effect for receivers by exploring the moderating role of desirable-feasible gift type. As we discussed in Study 2, low-priced gifts might indicate low use cost and emotional cost, consequently, receivers evaluated low-priced gifts as better than high-priced gifts. If this explanation held, we expected that the preference for the low-priced gift (vs. high-priced gift) would be demonstrated only for the feasible gift but not for the desirable gift, because only the feasible gift indicated low use cost. The desirable gift, which emphasized the high desirability and low feasibility, did not imply a low use cost; even for a low-priced desirable gift was still not associated with a lower use cost, therefore, the preference for the low-priced gift would not hold for the desirable gift.

### Methods

A total of 118 Chinese college students (66 males, 51 females, and 1 who did not report gender; *M*_*age*_ = 20.21 years, *SD* = 1.32) were randomly assigned into a 2 (gift price: low vs. high) × 2 (gift type: feasible gift vs. desirable gift) between-subjects design and instructed to imagine that they were receiving a gift, and to evaluate the gift. The Study 3B procedures were the same as those used in Study3A, except that all participants were assigned the role of the gift receiver.

### Results

#### Manipulation Check

Participants perceived that the feasible gift was more feasible (*M* = 4.68, *SD* = 1.02 vs. *M* = 3.75, *SD* = 1.54), *t*(115) = 3.87, *p* < 0.001, *d* = 0.71, and the desirable gift were perceived as more desirable (*M* = 4.79, *SD* = 1.46 vs. *M* = 3.67, *SD* = 1.30), *t*(115) = 4.40, *p* < 0.001, *d* = 0.81, showing that the gift type manipulation was successful. Moreover, the gift were perceived as more expensive by participants in the high price condition (*M* = 4.67, *SD* = 1.21) than those in the low price condition (*M* = 3.81, *SD* = 0.96), *t*(116) = 4.25, *p* < 0.001, *d* = 0.79, showing that the gift price manipulation was also successful.

#### Gift Evaluation

**FIGURE 5:**
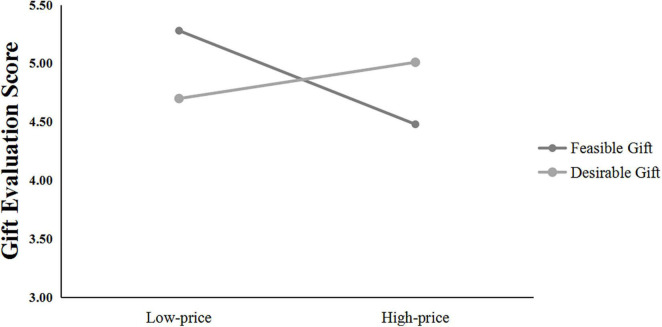
Study 3B: Gift evaluation score of gift receivers.

The average of the four gift-evaluation scores was the dependent variable (α = 0.90). A 2 (gift price: low vs. high) × 2 (gift type: feasible gift vs. desirable gift) ANOVA on the score showed that the main effects of gift price and gift type were not significant (*p*s > 0.05). More importantly, it yielded a significant interaction effect of gift type and gift price, *F*_(1, 114)_ = 5.70, *p* = 0.019, η*_*p*_*^2^ = 0.05, as shown in Figure 5, supporting the hypothesis. Further simple effect analysis showed that, for the desirable gift, the evaluation of high-priced gift (*M* = 5.01, *SD* = 1.13) did not differ significantly from the low-priced gift (*M* = 4.70, *SD* = 1.30), *p* = 0.327. However, for the feasible gift, the gift evaluation score of the low-priced gift (*M* = 5.23, *SD* = 1.14) was significantly higher than that of the high-priced gift (*M* = 4.48, *SD* = 1.25), *p* = 0.018, *d* = 0.63, which replicated the results of Study 2.

### Discussion

The results of Study 3A and 3B validated our predictions and identified the gift types as the boundary condition for the pricing effect, by showing that givers evaluated high-priced gifts as better only for the desirable gift, and receivers evaluated low-priced gift as better only for the feasible gift. Moreover, the results provided indirect evidence that our reasoning was right: givers focus more on the benefits (the “why” aspects of gift-giving, such as desirability and quality of the gift), and considered price as an indicator of quality; receivers were more sensitive to the cost (the “how” aspects of gift-giving, such as feasibility and emotional cost of the gift), and considered price as an indicator of cost. An interesting finding was that the high-priced desirable gift was regarded as the best gift from the perspective of givers, but the low-priced feasible gift was seen as the best gift by receivers. It seemed that there was a compatible pattern among givers-high-priced-desirable gift and receivers-low-priced-feasible gift, which actually reflected that givers were more concerned about the benefits of the gift and the receivers were more concerned about the cost which receivers themselves needed to bear (such as maintaining cost). If pricing is taken as the external value measure to influence consumers’ judgments, it only seems to affect gift givers and only for the desirable gift.

## General Discussion

The present research explored differences in gift evaluation between gift givers and receivers. Across three studies, the results showed that gift evaluations differed between givers and receivers, where givers inferred quality from price and believed that high price indicated good quality, and good quality indicated a good gift, especially for desirable gifts. By contrast, receivers evaluated feasible gifts as better than desirable gifts, did not infer quality or monetary sacrifice from price, and evaluated low-priced gifts as better than high-priced gifts, especially for feasible gifts. Our research contributes to the gift-giving literature by demonstrating the effect of pricing on gift evaluation; the findings could contribute to understand the differences between givers’ and receivers’ preferences regarding a “good gift” and the underlying psychological mechanism.

### The Influence of Pricing on Gift Evaluation and Its Mechanism

Previous studies have focused on the feelings of the receiver rather than on the evaluation of the gift itself. For example, Wang and Van der Lans (2018) used price as an indicator of the importance of the giver’s relationship with the receiver, and Flynn and Adams (2009) associated the higher price with givers’ higher level of thoughtfulness and receivers’ higher level of feeling appreciated. It seems that previous research supported the notion that “more expensive, more appreciative” rather than “more expensive, more attractive.” Moreover, the way we manipulated price in the present research was different from that of previous researchers, as they used different products as the target gift to manipulate the price, which might confound the effect of pricing. Specifically, previous researchers have explored the effect of expensive gifts (i.e., an iPod) and inexpensive gifts (i.e., a CD) on givers’ and receivers’ appreciation (Flynn and Adams, 2009). However, an identical product was used as the target gift in our research, to exclude the potential confounding effect of different products and investigate the effect of pricing on gift evaluation.

The findings showed that pricing affected gift evaluation, and that the impact of pricing on gift evaluation differed between givers and receivers. As expected, givers inferred quality from the price: high price meant high quality, and high quality meant a good gift. Although givers used price to infer monetary sacrifice as well, the perceived monetary cost did not influence their gift evaluations. Conversely, receivers unexpectedly and interestingly, evaluated low-priced gifts as better than high-priced gifts. They did not use price to infer quality or monetary cost and did not use monetary cost to evaluate gifts; the only inference was that good quality indicated a good gift, which suggested that price might influence gift evaluation through paths other than the perceived quality and perceived monetary cost. As we discussed in Study 2, the findings that receivers preferred a low-priced gift could be explained from the perspective of cost (use cost and emotional cost).

To summarize, givers prioritize the benefits of the gift, such as its desirability and its quality, and even when they feel the monetary cost, this barely influences their gift evaluation (Bornemann and Homburg, 2011; Baskin et al., 2014). By contrast, receivers seem to prioritize the cost of the gift, including the use cost (i.e., feasibility of the gift) and the emotional cost, but not the monetary cost which they do not need to bear. Givers tend to use price as a clue for the gift’s benefits, believing that “more expensive, more attractive,” while receivers tend to use price as a clue for the gift’s cost; consequently, givers prefer high-priced gifts and receivers prefer low-priced gifts, even when the gift is identical.

### The Boundary Condition for Price as a Good-Gift Clue

The desirable-feasible gift type was identified as the boundary condition for the pricing effect. Specifically, givers rated a high-priced gift as better than a low-priced gift when evaluating a desirable gift (but not a feasible gift), while receivers rated a low-priced gift as better than a high-priced gift when evaluating a feasible gift (but not a desirable gift). Givers appear to view high price as high quality for the desirable gift. This is in accordance with previous research which found that the stronger focus on desirability was the reason behind consumers viewing high price as an indicator of high quality when the psychological distance was far (Bornemann and Homburg, 2011). Specifically, the desirable gift description increased the consideration given by givers to the desirability, whereas the feasible gift description increased the consideration given by givers to the feasibility. Consequently, givers would see high price as high quality for the desirable gift, and the high-priced gift was evaluated as better than low-priced gift for the desirable gift. Therefore, the giver believes that the notion of “more expensive, more attractive” is limited to desirable gifts but not applicable to feasible gifts. Conversely, receivers might prioritize the cost, and view low-priced gifts as having low use and emotional costs, making them better gifts. The results showed the receivers’ preference for low-priced feasible gifts because these were associated with low use cost. Additionally, these gifts can also be seen as more thoughtful, meaning that givers invested more time and thought when choosing them (high behavioral cost) (Robben and Verhallen, 1994). Taken together, receivers would take low-priced feasible gift as the low use cost, low emotional cost and thoughtful gift, therefore a better gift. However, the desirable gift, which emphasized high desirability but low feasibility, was not a lower use cost gift; therefore, the preference for the low-priced gift would not hold for the desirable gift.

Additionally, when comparing the pricing effects of desirable gift and feasible gift between givers and receivers, an interesting finding indicated that the high-priced desirable gift was regarded as the best gift by givers, but the low-priced feasible gift was viewed as the best gift by receivers, which could be seen as evidence that givers prioritize the benefits of the gift; while receivers prioritize its cost. If pricing is taken as the external value measure that influences consumers’ judgments, it is only applicable to gift givers and only for the desirable gift.

### What Makes a Good Gift?

The present research could also contribute to understand what makes a good gift. Good gifts may have different dimensions. For example, a personal gift can be divided into a utilitarian gift or an expressive gift, where givers invest expressive gifts with greater symbolic value than utilitarian gifts, and utilitarian gift exchanges occur where role distance between partners is relatively far (Tournier and Gilmour, 1963). Similarly, previous research has shown that givers’ gift-giving motives can be divided into smile-seeking and value-seeking. Therefore, there are smile-seeking gifts that are superior on visceral attributes, such as a dozen blooming roses or a gift card redeemable immediately, and value-seeking gifts that have more balanced visceral and cerebral attributes that bring higher overall benefits, such as two dozen rose buds that can be enjoyed for longer, or a delayed gift card with a higher value (Yang and Urminsky, 2016). In fact, in addition to being regarded as a process of economic and social instrumental exchange, gift-giving can also be a process of expressing selfless love (Belk and Coon, 1993; Schiffman and Cohn, 2009). Consistent with this view, prior research also demonstrated that sentimental value is an important measure of a good gift (Givi and Galak, 2017), and the emotional response of givers and receivers is important in gift-giving (Taute and Sierra, 2015).

A recent review that summarized the gift preference difference between givers and receivers proposed that the different criteria for good gifts stemmed from gift givers focusing on the moment of exchange, while receivers focused on how valuable a gift is over the long run. Specifically, givers cared more about the emotional response of the receiver when the gift was opened, whereas receivers cared more about the long-term utilitarian value of the gift (Galak et al., 2016). Overall, it seems that a good gift should meet two criteria: it must meet the receiver’s utilitarian needs (i.e., the objective criterion of a useful and good quality product); and it must meet the receiver’s psychological needs (i.e., the subjective criterion of expressing thoughtfulness). As Zhang and Epley (2012) indicated that, other than the objective quality, receivers also felt appreciation and gratitude for the thought that went into the gift as an independent source of value, especially when a bad gift was given by a friend.

The present research also provided two criteria for good gifts from the perspective of both givers and receivers. For givers, a good gift should be of good utility and quality, therefore a high-priced desirable gift is the best gift; while for receivers, a good gift should be a care-free gift, with low usage cost and low emotional cost, therefore a low-priced feasible gift is the best gift. However, these two criteria do not contradict the above objective utilitarian criterion and subjective psychological criterion, for that good utility and quality could be included in the utilitarian criterion, and a care-free gift with low usage cost and emotional cost could reflect the gift givers’ thoughtfulness, which is consistent with the subjective psychological criterion. Furthermore, the present research demonstrated that the price and the desirability of the gift could be regarded as the measure of the objective utilitarian criterion, and suggested that the feasibility and price of the gift could be taken as the measure of the subjective psychological criterion. Specifically, givers regarded price as the objective quality criterion of a good gift, where high price indicated good quality, and good quality indicated a good gift. By contrast, receivers considered price as the subjective psychological criterion, where low price indicated thoughtfulness, and thoughtfulness indicated an intimate relationship with the giver, and a gift from an intimate friend is a good gift. Different from the opinion that receiver’s focused more on the objective utilitarian criteria of a good gift (e.g., Galak et al., 2016), we believe that receivers value, not only objective utilitarian criteria, but also subjective psychological criteria. When a gift comes from a friend, the thoughtfulness expression is very important as well. Sometimes givers realize the importance of expressing thoughtfulness, but they often fail to express it appropriately, for example, they tend to convey thoughtfulness by buying expensive gifts, although the receiver does not appreciate this the way the giver expected (Flynn and Adams, 2009).

### Practical Implications

In addition to the theoretical contributions, this research also has important practical implications both for consumers and marketing practitioners. Givers believe that a high-priced gift is better than a low-priced gift, especially for a desirable gift. In other words, they believe the notion “more expensive, more attractive.” It reminds us that, as gift givers, the consumers should avoid high-pricing marketing traps and select gifts more rationally, instead of regarding price as the reliable value label. By contrast, feasibility (i.e., usage convenience) matters more to receivers, who do not believe that high price necessarily means a good gift, and low price necessarily mean a bad one. It seems that the key is the thoughtfulness that the gift expressed, and thoughtfulness could not be expressed solely by gift price.

For gift marketers, first, high-pricing strategies should be adopted, not only to meet the marketers’ profit demands but also to conform to givers’ belief that “more expensive, more attractive.” Moreover, considering that the desirable gift is the boundary condition of pricing effect, gift retailers should focus on selling high-priced desirable gifts, such as luxury watch. Second, a good gift should also satisfy the receiver’s preferences for a “care-free gift” and thoughtful gift. For example, sellers could reduce the perceived cost of using gifts by providing free maintenance services. The maintenance fee could be paid by the giver through the packaging pricing: on the one hand, it could cover the maintenance cost; on the other hand, it could further increase the price of the gift, which would also improve the attractiveness of the gift from the givers’ perspective. To explicitly express the thoughtfulness of the gift giver, the gift’s brand should highlight a free maintenance service, and even directly express love and care for receiver on behalf of giver, in the wording used on the gift package. Additionally, the price of a gift should only be presented to givers rather than receivers, which could improve the attractiveness of the gift for givers without bringing too much emotional burden for receivers. Sometimes, words speak louder than money (Servátka et al., 2011).

Furthermore, understanding that the psychological distance difference and price-quality inference is the psychological mechanism behind the phenomenon of “more expensive, more attractive” is of special practical significance. For consumers, that would help them overcome this mindset. Specifically, consumers could choose the gift more rationally by reducing the psychological distance, rather than only focusing on the desirability and price of the gift. For example, they could imagine how themselves would use the gift as a gift receiver. For marketers, they should ensure that there is a far enough psychological distance between the consumer and the gift in order to make full use of the effect of “more expensive, more attractive.” For example, the psychological distance could be increased by showing in advertisements how a certain gift could contribute to the “ideal self” and the “ideal life,” or by displaying the product in a luxurious and unrealistic atmosphere to increase the psychological distance.

### Limitations and Future Research

Although this research obtained some interesting findings, it also has some limitations. First, the participants of this study were Chinese college students. They were not economically independent, and their consumption levels were relatively low (about 1,100 RMB per month on average, i.e., approximately $174). Moreover, previous studies have demonstrated cultural differences in price-quality inference, highlighting how the price influenced gift evaluation in different cultures (e.g., Lalwani and Shavitt, 2013; Lalwani and Forcum, 2016). Therefore, caution needs to be ensured when generalizing the conclusions of our study to other groups (e.g., high-income groups) or other cultures. A cross-cultural comparison might be an interesting direction for future research. Our research was conducted in China, and found that price was used as an indicator of a good gift by gift givers. The results were consistent with those of previous studies, which suggested that interdependent and high-power distance Chinese consumers were more likely to use price to infer product quality (Lalwani and Shavitt, 2013; Lalwani and Forcum, 2016). However, the western culture is more independent and has relatively low-power distance, so how pricing influences givers’ and receivers’ gift evaluations in western culture would be an interesting research question.

Second, the occasion of birthday gift-giving between friends was adopted in our study based on the results of the pretests, which was suitable for the participants (Chinese college students). However, prior research has demonstrated that the gift-giving occasion, such as business gift-giving (Baskin et al., 2014), the relationship between giver and receiver, such as superior subordinate relationship (Choi et al., 2018), romantic relationship (Belk and Coon, 1993), etc., and even the sex of givers and receivers (Saad and Gill, 2003) could influence gift evaluations. Therefore, whether the conclusion of the present research could be extended to other gift-giving contexts, needs to be addressed by the future research.

Third, our research focused on the effect of pricing on the gift evaluations of givers and receivers and its underlying psychological mechanism. The results supported our predictions that the gift giver considered that “more expensive, more attractive” and the price quality inference was the underlying mechanism; The results also unexpectedly showed that gift receivers preferred low-priced gifts, especially for low-priced feasible gifts, and that the dual roles of price were not the underlying mechanism. However, these conclusions are actually drawn based on low-end product (i.e., earphone) which suitable for college students. Therefore, whether using high-end products (thanks for the reviewer’s enlightening comment) and other types of products as target product will draw the consistent conclusion remains to be tested by future research.

We also noticed that the effect sizes of some results are medium or even small, especially for the results of the receiver’s preference for low-priced gift (thanks for the reviewer’s enlightening comment). However, the effect size of the results that supports the giver’s preference for high-priced gift is rather large. These results suggested that the effect of pricing on gift evaluation for givers is more stable, while the effect of pricing on evaluation for receivers is vulnerable which suggested that the receiver’s evaluation of gifts is also affected by other factors, and its mechanism needs to be further explored. We reason that the receivers’ preference for low-priced feasible gifts stemmed from their sensitivity to cost and their preference for care-free gifts, but this explanation need direct empirical evidence. Future research could explore the influence of cost-related factors on gift evaluation and its underlying mechanism, which would help to further understand the good gift criterion for givers and receivers.

## Conclusion

There are differences in gift evaluation between givers and receivers. Specifically, givers evaluate the high-priced gift as better than the low-priced gift, and the “more expensive, more attractive” effect is supported. Givers infer quality from price, and believe that high price indicates good quality, and good quality indicates a good gift, especially for a desirable gift. Receivers evaluate a feasible gift as better than a desirable gift, they do not use price as an indicator of quality or monetary cost. “More expensive, more attractive” does not resonate with them, on the contrary, they evaluate a low-priced gift as better than a high-priced gift, especially for a feasible gift.

## Data Availability Statement

The raw data supporting the conclusions of this article will be made available by the authors, without undue reservation.

## Ethics Statement

The studies involving human participants were reviewed and approved by the Institutional Review Board of the School of Psychology at the Shandong Normal University. The patients/participants provided their written informed consent to participate in this study.

## Author Contributions

NL contributed to the conception, design of the research, data analysis, and original draft writing, reviewing and editing. YL contributed to original draft writing and data analysis, performed the experiments. XW performed the experiments. SL contributed to reviewing and editing. All authors contributed to the article and approved the submitted version.

## Conflict of Interest

The authors declare that the research was conducted in the absence of any commercial or financial relationships that could be construed as a potential conflict of interest.

## Publisher’s Note

All claims expressed in this article are solely those of the authors and do not necessarily represent those of their affiliated organizations, or those of the publisher, the editors and the reviewers. Any product that may be evaluated in this article, or claim that may be made by its manufacturer, is not guaranteed or endorsed by the publisher.

## Footnotes

1 Forty participants participated in the pretest to validate the earphone descriptions. When the earphones were described as a feasible gift, participants indeed considered them as more feasible (*M* = 5.43 vs. *M* = 3.23), *t*(39) = 8.27, *p* < 0.001; when described as a desirable gift, participants perceived the earphones as more desirable (*M* = 5.05 vs. *M* = 3.80), *t*(39) = 4.24, *p* < 0.001.
